# Outbreak of *Salmonella* Chailey Infections Linked To Precut Coconut Pieces — United States and Canada, 2017

**DOI:** 10.15585/mmwr.mm6739a5

**Published:** 2018-10-05

**Authors:** Sarah Luna, Marsha Taylor, Eleni Galanis, Rod Asplin, Jasmine Huffman, Darlene Wagner, Linda Hoang, Ana Paccagnella, Susan Shelton, Stephen Ladd-Wilson, Sharon Seelman, Brooke Whitney, Elisa Elliot, Robin Atkinson, Katherine Marshall, Colin Basler

**Affiliations:** ^1^Epidemic Intelligence Service, CDC; ^2^British Columbia Centre for Disease Control, Vancouver, British Columbia; ^3^Fraser Health Authority, Delta, British Columbia; ^4^Division of Foodborne, Waterborne, and Environmental Diseases, CDC; ^5^IHRC Inc., Atlanta, Georgia; ^6^Washington State Department of Health; ^7^Oregon Health Authority; ^8^Food and Drug Administration, Washington, DC; ^9^Canadian Food Inspection Agency, Ottawa, Ontario.

Foodborne salmonellosis causes an estimated 1 million illnesses and 400 deaths annually in the United States (*1*). In recent years, salmonellosis outbreaks have been caused by foods not typically associated with *Salmonella*. On May 2, 2017, PulseNet, CDC’s national molecular subtyping network for foodborne disease surveillance, identified a cluster of 14 *Salmonella* Chailey isolates with a rare pulsed-field gel electrophoresis (PFGE) pattern. On May 29, Canadian health officials informed CDC that they were also investigating a cluster of five *Salmonella* Chailey infections in British Columbia with the same PFGE pattern. Nineteen cases were identified and investigated by CDC, U.S. state health departments, the Public Health Agency of Canada, and the British Columbia Centre for Disease Control. Isolates from all cases were highly related by whole genome sequencing (WGS). Illness onset dates ranged from March 10 to May 7, 2017. Initial interviews revealed that infected persons consumed various fresh foods and shopped at grocery chain A; focused questionnaires identified precut coconut pieces from grocery chain A as a common vehicle. The Canadian Food Inspection Agency (CFIA) and the U.S. Food and Drug Administration (FDA) conducted a traceback investigation that implicated a single lot of frozen, precut coconut as the outbreak source. Grocery chain A voluntarily removed precut coconut pieces from their stores. This action likely limited the size and scope of this outbreak.

## Epidemiologic Investigation

A case was defined as infection with *Salmonella* Chailey with the outbreak PFGE pattern with illness onset during March 10–May 7, 2017, and highly related by WGS to other cases. Nineteen cases were identified: 14 in seven U.S. states (one case each in Colorado and Kansas, two each in Oregon, Pennsylvania, Utah, and Washington, and four in Texas) and five cases in British Columbia, Canada (Figure). Infected persons ranged in age from <1 to 87 years (median = 57 years), including two aged <5 years; nine persons were female. Among 17 persons for whom information on hospitalization was known, three were hospitalized; no deaths occurred.

**FIGURE:**
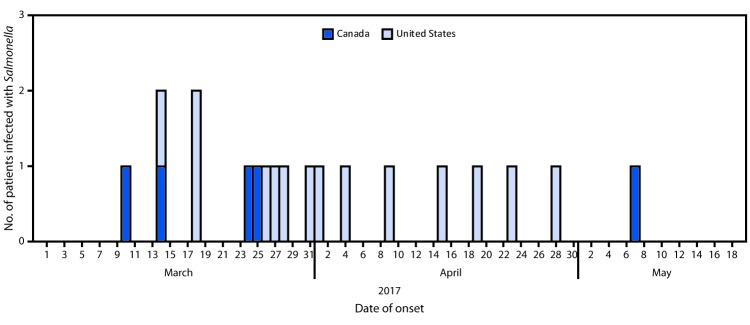
Number of persons infected with the outbreak strain of *Salmonella* Chailey (N = 19), by date of illness onset — United States and Canada, 2017

Infected persons in the United States were initially interviewed using state-developed questionnaires or CDC’s National Hypothesis Generating Questionnaire; both collected information on foods consumed and locations where food was purchased during the 7 days before illness onset. Review of data collected using these questionnaires revealed that among nine persons with information on grocery stores, seven reported shopping at grocery chain A, which comprises health food stores. Other commonly reported foods consumed included oranges (six persons), strawberries (five), tomatoes (four), kale, tuna, zucchini, almonds (three each), and shrimp (two). The tuna and other seafood exposures were noteworthy because a strain with the outbreak PFGE pattern had been isolated from yellowfin tuna imported from Indonesia in 2010. Because of the strong fresh-foods signal from the initial information, open-ended interviews were conducted to obtain more information about foods purchased from grocery chain A and other fresh foods that were not included on the standard National Hypothesis Generating Questionnaire (*2*) used during the initial interviews. Open-ended, iterative interviews were conducted by a single interviewer to gather more detailed information about foods persons ate before they became ill. Interviews were completed for eight persons, including five who had already been interviewed with a standard questionnaire. One person reported eating precut coconut pieces from grocery chain A, two persons reported drinking coconut water, two reported eating sushi, seven reported eating oranges, and three reported eating seaweed snacks. Because open-ended interviews did not identify a single food item of interest, a focused questionnaire was developed. The focused questionnaire included detailed, open-ended questions about food items purchased from grocery chain A, as well as specific questions asking about consumption of coconut, coconut water, other fruits, vegetables, nuts, seaweed, sushi, and other fish.

At the same time, Canadian investigators used a centralized interviewer approach to interview all five infected persons in Canada using a modified version of CDC’s focused questionnaire. All five persons reported shopping at grocery chain A locations in Canada and consuming precut coconut pieces purchased there. Eleven infected persons in the United States were reinterviewed with the focused questionnaire, and six reported eating precut coconut pieces from grocery chain A. In total, 16 persons in the United States and Canada were reinterviewed, and 11 reported consuming precut coconut pieces from grocery chain A.

CDC and the British Columbia Centre for Disease Control requested consumer purchase information from grocery chain A to continue to generate hypotheses while reinterviewing persons. Because grocery chain A did not have a shopper card program, consenting persons were asked to share the purchase dates, total purchase dollar amounts, store location, and the first six digits and last four digits of the credit card used at time of purchase. Grocery chain A used this information to retrieve receipts.

Seven persons provided information to retrieve receipts from six grocery chain A locations in British Columbia, Oregon, and Texas. Receipts were retrieved for all seven persons, four of whom (one person in the United States, who initially did not report coconut exposure, and three persons in Canada) had precut coconut pieces listed on their receipts (purchase dates March 7–15, 2017). Another person who did not provide information to retrieve receipts reported purchasing precut coconut pieces on April 13. A total of 12 persons reported eating precut coconut pieces or had receipts verifying the purchase of precut coconut pieces from grocery chain A.

## Laboratory Investigation

Clinical isolates were characterized by WGS. Whole genome, high-quality single nucleotide polymorphism (hqSNP) analysis* indicated that the 19 clinical isolates differed by 0–4 hqSNPs, indicating high genetic relatedness. An additional two *Salmonella* Chailey isolates with the same PFGE pattern from persons in the United States and Canada with illness onset dates consistent with this outbreak were excluded, as they differed from the rest of the isolates by approximately 100 hqSNPs. The isolates from yellowfin tuna imported from Indonesia in 2010 were 19 hqSNPs different from the clinical isolates and were also considered to be not closely related genetically.

## Inspections and Traceback

Canadian officials conducted an inspection at a location of grocery chain A and reported that frozen, vacuum-packed coconut pieces were received at the store every other day. These were thawed at the store and repacked into smaller plastic tubs for sale in the produce area, with a 5-day shelf life applied. Grocery store A headquarters communicated to U.S. officials that all of their stores thaw and repack this product in the store. FDA visited three U.S.-based, FDA-regulated firms associated with the import and repackaging of this product and identified no objectionable conditions.

CFIA and FDA conducted a traceback investigation for nine persons in the United States and Canada who all reported consuming precut coconut pieces sold by grocery chain A. These locations received product from three distribution centers located in three states that obtained frozen precut coconut pieces from the same U.S. firm. Records collected by FDA and CFIA at grocery chain A locations, distribution centers, and the processor suggested that a single lot of frozen precut coconut pieces imported from Indonesia was the outbreak source. FDA tested environmental and coconut samples from processing and distribution centers, but no *Salmonella* was detected. However, coconut from the suspected lot was not available for testing.

## Public Health Response

Based on the results of the epidemiologic investigation, grocery chain A voluntarily removed thawed, precut coconut pieces from store shelves, which included all precut coconut pieces from the lot identified by the traceback investigation. No public communication was issued, given that this action, combined with the 5-day shelf life of thawed precut coconut pieces, made it unlikely that contaminated precut coconut pieces were still available for purchase or in customers’ homes.

## Discussion

International collaboration on the epidemiologic and laboratory investigation was important for identifying that the Canadian and U.S. cases were part of the same cluster. This allowed investigators to focus on food purchased at grocery chain A and to identify frozen precut coconut pieces as the outbreak source.

Early communication and collaboration with grocery chain A assisted the investigation through the collection of detailed purchase history information and facilitated a rapid removal of precut coconut from stores. The timely action of grocery chain A likely limited the size and scope of this outbreak.

In recent years, salmonellosis outbreaks have been caused by foods not typically associated with *Salmonella*. This was the first time that coconut has been associated with an outbreak of *Salmonella* in the United States or Canada (*3*). Cases were reported throughout the United States and Canada that were associated with different grocery chain A locations, supplied by different distribution centers. The single lot of imported, precut coconut pieces was processed over many months but remained frozen and minimally manipulated once in the United States. Therefore, contamination likely occurred in the country of origin, Indonesia. Furthermore, the frozen yellowfin tuna with the same PFGE pattern was imported from Indonesia in 2010, providing support for the hypothesis that a food product from Indonesia could be the source of the outbreak.

This was a complicated investigation, and it required considerable time and effort by investigators in two countries to identify the food product ultimately responsible for the outbreak. Although no coconut from the suspected lot was available for laboratory sampling, epidemiologic and traceback information indicates that frozen precut coconut pieces were the source of the outbreak. In light of this finding, public health officials might consider raw coconut in investigations of *Salmonella* outbreaks among consumers of fresh foods.

**Summary**

**What is already known about this topic?**

Foodborne salmonellosis causes an estimated one million U.S. illnesses and 400 deaths annually.

**What is added by this report?**

During March–May 2017, an outbreak of 19 cases of *Salmonella* Chailey associated with precut coconut pieces from a single grocery store chain occurred in the United States and Canada. The chain voluntarily recalled precut coconut pieces. This was the first time that coconut has been associated with a *Salmonella* outbreak in the United States or Canada.

**What are the implications for public health practice?**

In recent years, salmonellosis outbreaks have been caused by foods not typically associated with *Salmonella*. Raw coconut should now be considered in investigations of *Salmonella* outbreaks among fresh food consumers.

## Footnotes

* Whole genome, high-quality single nucleotide polymorphism analysis was performed using the Lyve-SET hqSNP pipeline. https://github.com/lskatz/lyve-SET.
